# EU HTA Regulation and Joint Clinical Assessment—Threat or Opportunity?

**DOI:** 10.3390/jmahp12020008

**Published:** 2024-05-13

**Authors:** Volker Schuster

**Affiliations:** SAI MedPartners, Reading, PA 19606, USA; vschuster@sai-med.com

**Keywords:** EU Health Technology Assessment (HTA), Joint Clinical Assessment (JCA), European Network for Health Technology Assessments, (EUnetHTA), EU HTA regulation, PICO scheme, HTA Coordination Group (HTACG), JCA dossier, JCA submission

## Abstract

The vision of a unified European HTA is by no means a new endeavor. At its core are the publicly declared ambitions to harmonize assessments of clinical data within the EU and avoid the duplication of efforts. Not surprisingly, these ambitions are publicly announced to be motivating the new 2022 EU HTA regulation. However, industry experts typically see more of a risk for additional bureaucracy resulting in delays, further scrutiny, and one additional EU (clinical) dossier to submit on top of all national HTA dossiers, which could be considered a duplication of effort and therefore counterproductive. Regardless of how the details of the process will be defined and how the entire process will work in practice, we can be sure that EU officials will refer to the EU HTA and Joint Clinical Assessment (JCA) in particular as a learning system. The purpose of this article is to take a closer look at the new EU HTA regulation and analyze threats and opportunities for manufacturers and what the resulting opportunities and threats will be at the affiliate level throughout the EU.

## 1. Introduction

For years, industry experts have looked with some apprehension at the voluntary collaboration of EU member states through the EUnetHTA Joint Action and its predecessors. In December 2021, the EU finally passed a regulation toward a more formal collaboration with respect to HTA throughout the EU member states [1].

The regulation provides the legal basis for a permanent and sustainable cooperation between EU HTA bodies at the EU level. According to the regulation, this cooperation consists of the following four main topics:

- Joint clinical assessments (JCA);
- Joint scientific consultations (JSCs), whereby medicine manufacturers can seek advice from HTA bodies and regulators;
- Horizon scanning;
- Continuing voluntary cooperation in areas not covered by joint clinical assessments in which individual EU countries will continue to be responsible (non-clinical aspects of health technologies, decisions on pricing and reimbursement).

Here, we will focus on the JCA, which focuses on the clinical part of HTA, in particular relative efficacy/effectiveness and relative safety. All other aspects of the HTA process, especially the economic evaluation, will remain the responsibility of the national HTA bodies of the individual member states.

## 2. The HTA Regulation and Its Impact on Manufacturers

The HTA regulation 2021/2282 [1] emphasizes the fact that, in accordance with Article 168 (7) of the Treaty of the Functioning of the EU, “member states are responsible for the definition of their health policy and delivery of health services and medical care”. Also, the above HTA regulation clarifies that member states are the decision-makers with respect to “allocation of resources”, resulting in the need to restrict EU involvement in the HTA to the “aspects of HTA that relate to the joint clinical assessment” [1]. The individual member states are and fully remain budget holders, the decision-makers on affordability and availability.

The JCA is meant to be a “scientific analysis of the relative effects of the health technology”, including the “degree of certainty of the relative effects, taking into account the strengths and limitations of the available evidence” [1]. In accordance with Article 168 (7) of the Treaty of the Functioning of the EU, there are “no value judgments” in the JCA. This is only consistent with Article 168 (7) of the Treaty of the Functioning of the EU, which leaves member states the authority and responsibility to define their own health policies, including, but not limited to, the allocation of funds.

The JCA process is managed by the HTA Coordination Group (HTACG) [1]. The members of the HTACG are representatives of the member states’ HTA bodies. The JCA process is set in motion when the HTACG appoints two rapporteurs, who need to come from HTA bodies of different member states. The rapporteurs are responsible for the clinical assessment, drafting a report, and consulting with stakeholders.

The two rapporteurs conduct a scoping process to define the research question and scope of the entire assessment in the form of PICO (population, intervention, comparator, outcomes) scheme(s) [2]. To do so, a PICO survey is sent to all 27 EU countries. Each country reports the PICOs required for its decision-making. The reported PICOs are consolidated by the two rapporteurs during the scoping process, and the manufacturer is not involved. Due to varying informational requirements of member states, the number of PICOs after consolidation can be expected to be more than one, and hence, go beyond the head-to-head evidence available at the time of launch.

The manufacturer has to prepare the JCA dossier after the end of the PICO survey and consolidation at day 120 of the regulatory EMA procedure. Roughly 100 days later, the manufacturer has to submit its JCA dossier, which needs to acknowledge all PICOs identified through scoping.

**Figure 1:**
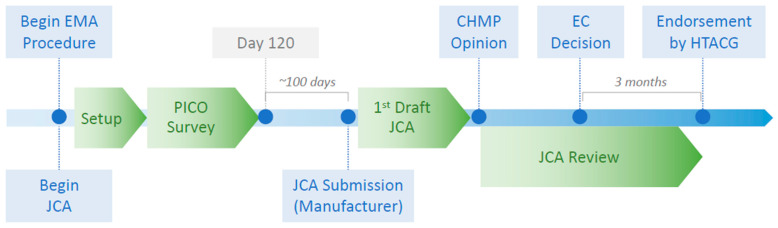
Above: General process overview of the Joint Clinical Assessment.

The first draft of the JCA is expected to be ready at the time of the Committee for Me-dicinal Products for Human Use’s (CHMP) opinion. Approximately three months after the European Commission’s (EC) decision, the JCA process will be finalized by the HTACG. According to the HTA regulation, the HTACG “should make all efforts to endorse the joint clinical assessment report by consensus” [1] (Figure 1). In cases wherein consensus cannot be reached, “divergent scientific opinions should be included” in the report.

From the time the required PICOs are decided, the manufacturer has little more than three months to submit its JCA dossier. This dossier will consist of the following six parts [3,4]:

1. Overview: administrative information and executive summary;
2. Background: includes a characterization of the disease or medical condition to be treated or diagnosed, a description of the compound or treatment, and states the conclusion of the joint scientific consultation related to the JCA;
3. Research question and scope: the scope of the assessment needs to be defined;
4. Methods: the criteria for the selection of studies need to be defined, and the process for the retrieval and selection of relevant studies needs to be documented;
5. Results: this section starts with the documentation of the results of the information retrieval process documented in Part 4. It then characterizes the included studies and finally analyzes these results with respect to relative efficacy/effectiveness and relative safety;
6. References and appendices.

This structure is similar to the structure of the German “Arzneimittelmarktneuordnungsgesetz” (AMNOG) dossier. A dossier for the consolidated PICO requirements of all member states has to be developed in half or one-third of the time that should be planned for the German PICO requirements alone. As there is a risk that the number of required PICOs may even exceed five, it seems necessary for the manufacturer to prepare first JCA dossier drafts for all relevant PICOs reported by the member states.

Furthermore, the high number of required PICOs may make the availability of head-to-head evidence for most of them unlikely. Where no head-to-head evidence is available, indirect treatment comparisons (ITCs) need to be prepared in advance. While, even before the JCA, member states had differing requirements and asked for different PICOs, the new demand is for all these PICOs to be accounted for even before launch. Before the JCA, manufacturers typically only needed to worry about the PICOs of the select few first launch countries at this stage, and they had many months and sometimes even years to finally decide how to address the evidence gaps with respect to the PICO desires of the remaining countries. Also, while at the time of JCA submission ITCs may be the only means to comply with the PICO requests, at the time of launch in later-launch countries, there may be alternative data sources available, e.g., RWE from first-launch counties or new clinical trials. The required workload for the manufacturer’s JCA dossier will be considerable. In theory, the same amount of work may have been required anyway, albeit not all by the time of the JCA submission, but over the course of typically several years. In addition, member states’ HTA templates and evidence requirements continue to differ. Instead of harmonizing HTA templates at least wherever evidence requirements are similar, manufacturers are presented with one more HTA template, which is not really harmonized with member state HTA templates, and asks for the clinical part for not one, but all PICOs at the time of JCA submission. To ensure a timely submission, a considerable number of different PICOs need to be accounted for, and for each, a rather ambitious scientific analysis—including an indirect treatment comparison—needs to be prepared. Smaller biotechs with limited resources may have to decide not to launch within the EU because they may not be able to handle the resulting workload within the tight timeline of approximately three months.

## 3. Discussion

The clinical additional benefit assessment of the JCA is non-binding [1]. However, member states need to give the JCA report “due consideration”, which requires them to acknowledge and consider each JCA report that is available at the time of local HTA decision-making [1]. If the JCA report should not be available “at the time when the national HTA is finalized”, the member state can proceed without delaying the national HTA process [1].

According to the HTA regulation, manufacturers cannot be asked to submit the data for the JCA again for any national HTA [1]. In theory, this should reduce efforts at the national level. However, in practice, national dossier templates require the requested information in a number of different ways. Presently, it seems overly optimistic to assume that the JCA template will cover all the informational needs regarding the clinical part of the assessment of all member state HTA bodies.

Any national decision-making—even with respect to clinical benefits—still remains at the member state level. Consequently, member states will be allowed to perform complementary clinical analyses regardless of the availability of a JCA report, if such analyses are needed within their national HTA framework [1]. For the same reason, the member states are still allowed to apply a different methodology than that of the JCA report. Furthermore, member states may want to consider different patient populations, comparators, or endpoints than those included in the JCA report, and they are within their rights to request the respective information. Hence, manufacturers can expect to still be required to submit additional clinical data or prepare additional clinical analyses to meet HTA requirements at the member state level, and the reduction in affiliate-level HTA workload can be expected to be rather limited in many cases.

However, the JCA also leads to obligations for the member states and their HTA bodies. Member states shall provide the HTACG with information on the national HTA within 30 days of its completion, and they also need to “provide information on how the joint clinical assessment reports have been considered when carrying out a national HTA” [1]. The acknowledgement of the joint clinical assessment reports at the member state level needs to be published. While the national HTA is not required to come to the same conclusion as the EU JCA and may lead to a different conclusion with respect to clinical effectiveness/efficacy and safety, the local HTA needs to report the difference, and moreover, needs to provide a rationale that is fully in line with present state-of-the art science. In cases wherein the JCA itself states “differences in opinion” or where no unambiguous state-of-the-art scientific knowledge even exists, this may be easy to accomplish. Also, if the locally used PICO scheme deviates from the PICO scheme of the JCA, coming to a different clinical conclusion and providing a scientific rationale for it may be rather straightforward. In cases wherein the PICO scheme relevant to the member state has been covered by the JCA report and no “difference in opinion” exists, it may be a challenge for the member state’s HTA body to provide a scientific rationale for drawing a different conclusion at the member state level. In case such a scientific rationale cannot be provided adequately, the JCA report may invite the manufacturer to challenge the conflicting HTA decisions at the member state level legally.

## 4. Conclusions

While the JCA may increase the workload in European market access, prolong the time to market of life-saving compounds, or even prevent innovations from reaching EU patients at all, the JCA report may be leveraged to hold member state HTA bodies to more rigorous scientific standards, and hence, improve the predictability of HTA decision-making throughout the EU.

The better the regulators and member state HTA bodies can agree on the required PICOs, the less duplication of efforts and additional resources will be required, and consequently, the more life-saving innovations will reach EU patients. More and earlier stakeholder engagement of the manufacturers with member state HTA bodies and much better alignment with respect to clinical HTA standards among the member state decision-makers will be needed to ensure that meaningful and potentially life-saving innovations can reach EU patients. It is not realistic for a JCA dossier to be written within 100 days, so up-front preparation, combined with skillful anticipation of the right PICOs, will be required on the part of the manufacturer.

The burden on manufacturers is very high. It needs to be anticipated that—especially in the beginning—not all manufacturers will be able to submit their JCA dossiers on time. In such cases, it seems possible that a few national HTA dossiers for first-launch countries are submitted before the JCA process can be finalized. In order to ensure that life-saving treatments are not delayed in reaching patients in need, the member state HTA bodies may(if necessary) need to use their authority to decide without the JCA report at their disposal if the national HTA process would otherwise be prolonged.

Similar to the AMNOG system, the JCA will be a learning system. Presently, requirements are not fully clear, and experiences and learnings do not yet exist—neither on the manufacturer side nor on the payer side. Missing confidence on either side is going to make effective communication and exchange between all stakeholders challenging. It will be precisely this communication and exchange that member state HTA bodies need to excel at to align on requirements among themselves and to advise manufacturers in a fair, helpful, and reliable way to help them anticipate requirements very early and thereby empower them not to let patients down. Needless to say, manufacturers that want to deliver their innovations to patients need to become equally excellent at communication. They also have to become wizards at planning and anticipation.
